# ABC transporter activity linked to radiation resistance and molecular subtype in pediatric medulloblastoma

**DOI:** 10.1186/2162-3619-2-26

**Published:** 2013-10-04

**Authors:** Wendy J Ingram, Lisa M Crowther, Erica B Little, Ruth Freeman, Ivon Harliwong, Desi Veleva, Timothy E Hassall, Marc Remke, Michael D Taylor, Andrew R Hallahan

**Affiliations:** 1 Queensland Children’s Medical Research Institute, The University of Queensland, Brisbane, QLD, Australia; 2 Division of Oncology, Children’s Health Queensland, Royal Children’s Hospital, Brisbane, QLD, Australia; 3 Division of Neurosurgery, Arthur and Sonia Labatt Brain Tumor Research Centre, Program in Developmental and Stem Cell Biology, Hospital for Sick Children, University of Toronto, Toronto, ON, Canada; 4 QCMRI, Level 4, Foundation Building, Royal Children’s Hospital, Herston Road, Herston, Qld 4029, Australia

**Keywords:** Medulloblastoma, Radiation resistance, ABC transporter, ABCG2, ABCA1, Stem, Brain tumor, Verapamil, Reserpine, Sonic hedgehog

## Abstract

**Background:**

Resistance to radiation treatment remains a major clinical problem for patients with brain cancer. Medulloblastoma is the most common malignant brain tumor of childhood, and occurs in the cerebellum. Though radiation treatment has been critical in increasing survival rates in recent decades, the presence of resistant cells in a substantial number of medulloblastoma patients leads to relapse and death.

**Methods:**

Using the established medulloblastoma cell lines UW228 and Daoy, we developed a novel model system to enrich for and study radiation tolerant cells early after radiation exposure. Using fluorescence-activated cell sorting, dead cells and cells that had initiated apoptosis were removed, allowing surviving cells to be investigated before extensive proliferation took place.

**Results:**

Isolated surviving cells were tumorigenic *in vivo* and displayed elevated levels of *ABCG2*, an ABC transporter linked to stem cell behavior and drug resistance. Further investigation showed another family member, *ABCA1*, was also elevated in surviving cells in these lines, as well as in early passage cultures from pediatric medulloblastoma patients. We discovered that the multi-ABC transporter inhibitors verapamil and reserpine sensitized cells from particular patients to radiation, suggesting that ABC transporters have a functional role in cellular radiation protection. Additionally, verapamil had an intrinsic anti-proliferative effect, with transient exposure *in vitro* slowing subsequent *in vivo* tumor formation. When expression of key ABC transporter genes was assessed in medulloblastoma tissue from 34 patients, levels were frequently elevated compared with normal cerebellum. Analysis of microarray data from independent cohorts (n = 428 patients) showed expression of a number of ABC transporters to be strongly correlated with certain medulloblastoma subtypes, which in turn are associated with clinical outcome.

**Conclusions:**

ABC transporter inhibitors are already being trialed clinically, with the aim of decreasing chemotherapy resistance. Our findings suggest that the inhibition of ABC transporters could also increase the efficacy of radiation treatment for medulloblastoma patients. Additionally, the finding that certain family members are associated with particular molecular subtypes (most notably high *ABCA8* and *ABCB4* expression in Sonic Hedgehog pathway driven tumors), along with cell membrane location, suggests ABC transporters are worthy of consideration for the diagnostic classification of medulloblastoma.

## Background

Tumors of the central nervous system (CNS) are the leading cause of cancer related childhood death and disability in developed countries [1]. Of the malignant pediatric brain tumors, medulloblastoma is the most common and treatment typically involves surgery, radiotherapy and chemotherapy [2]. Age of onset, degree of surgical removal and presence of metastatic disease provide useful criteria to stratify patients into “standard” and “high risk” groups, however a subset of patients in both groups are prone to treatment resistance and relapse. Although recent studies show medulloblastoma patients cluster into at least four distinct groups based on gene expression signatures, it is not yet possible to determine which individuals will display treatment resistance [3].

Evidence suggests that increased resistance to both chemotherapy and radiation is a feature of Cancer Stem-like Cells (CSCs): the subset of cells within a tumor that have the ability to drive tumor re-growth or to initiate a metastatic lesion [4-7].

Resistance to radiation can arise from a variety of mechanisms, including enhanced DNA repair, altered apoptotic responses, tolerance of genomic instability and dormancy [8]. A key mechanism by which cells resist chemotherapy drugs is via ATP-binding cassette (ABC) transporters. The ABC transporter gene family has 50 members in humans, encoding membrane-bound pumps that transport an assortment of substrates [9]. Four members of this family have notable roles in multi-drug resistance: *ABCG2* (also known as *BCRP*), *ABCB1* (*Pgp*/*MDR1*), *ABCC1* (*MRP1*) and *ABCC2* (MRP2) [10,11]. *ABCG2* is of special interest, as it marks stem cells in a wide range of normal tissues, including brain, and may have a functional role in maintaining a non-differentiated state [12]. *ABCG2* is also implicated as a CSC marker in diverse malignancies [13,14].

While definitive markers of CSCs and resistant cells remain elusive, genes other than *ABCG2* are also expressed in tumor sub-populations enriched for stem-like behavior. These include other cell surface molecules (e.g. *prominin 1* (*CD133*), *FUT4* (*CD15*)); filaments (e.g. *nestin*); regulators of DNA architecture (e.g. *BMI1*) and mediators of developmental signaling networks (e.g. genes involved in the NOTCH, WNT and Sonic Hedgehog (SHH) pathways) [15,16]. Despite mounting evidence for the existence of CSCs in brain tumors, markers in medulloblastoma remain poorly understood [17,18].

Evidence of increased radiation resistance in brain CSCs has come mainly from studies of glioma and atypical teratoid/rhabdoid tumors (AT/RT) for which experimental isolation depended on CD133 [19-23]. Selection of CD133 positive cells enriches for CSCs in some tumors, but is not without controversy [17,24,25]. Nonetheless, activation of the DNA damage response and chromatin remodeling appear to mediate radiation tolerance in glioma [19,20,22]. A glioma study using a non-*CD133* driven experimental approach found Hedgehog pathway signaling important for maintaining radiation tolerant CSCs [26]. However, radiation resistance and its relationship to stem-like behavior has been much less studied in medulloblastoma. CD133 positive cells from the Daoy line are reported to have increased radiation tolerance, while *in vivo* work has shown Nestin expressing medulloblastoma cells to have enhanced survival after irradiation [27,28].

We undertook the reverse approach of many studies to examine radiation tolerant medulloblastoma cells. Rather than isolating cells with a putative marker and then investigating resistance, we turned the problem around by selecting surviving cells functionally, followed by a candidate gene approach to see if putative stem cell markers were associated with the radiation tolerance phenotype. This gave us the potential to identify characteristics present before radiation was encountered, as well as responses that might be preferentially up-regulated by cells after radiation exposure. This approach proved fruitful, with the discovery of several genes (including *ABCG2*), that were elevated in surviving cells. Furthermore, functional experiments indicated that ABC transporters may play a previously undiscovered role in protecting brain tumor cells from radiation induced death.

## Results

### Development of a model system for early enrichment of radiation tolerant tumor cells

**Figure 1:**
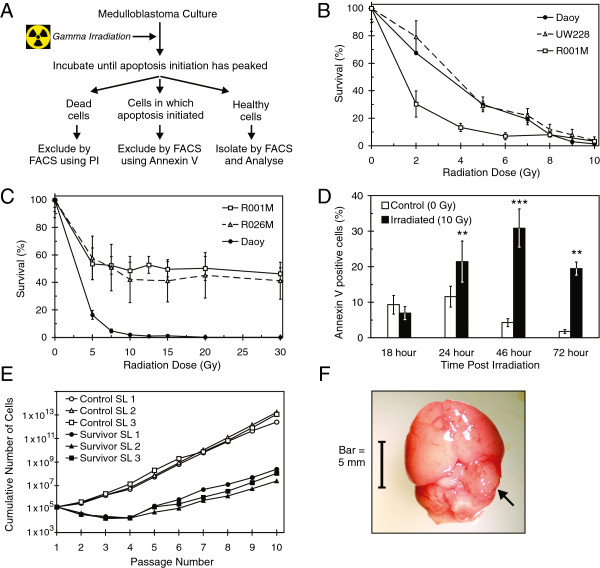
**Post-radiation survivor selection model yields tumor initiating cells. (A)** Outline of technique used for the early isolation of medulloblastoma sub-populations enriched for the most radiation tolerant cells. The method uses Propidium Iodide (PI) and Annexin V to exclude sensitive cells via Fluorescence Activated Cell Sorting (FACS). **(B)** Radiation dose response curves for UW228, Daoy and R001M, generated by Clonogenic Colony Forming Assay (which only includes actively proliferating cells). **(C)** Radiation dose response curves for Daoy, R001M and R026M, generated by resazurin based viability assay (measures all cells that have not yet died). **(D)** Profile of apoptosis initiation over time after irradiation of Daoy cells, detected by Immunofluorescence using Annexin V (240 to 1700 cells assessed per treatment, over 3 to 7 wells). Error bars in panels **B** to **D** represent +/− 1 SD. “**” = p-value < 0.01, “***” = p-value < 0.001, for control compared with irradiated cells. **(E)** Tumorsphere culture of Daoy cells surviving 10 Gy radiation. After an initial lag, independent survivor sub-lines (SLs) yield proliferative sphere cultures, which like non-irradiated (control) SLs, are able to be proliferated long term (spheres were passaged weekly). **(F)** Medulloblastoma cells shown in panel **E**, that survived 10 Gy, initiated large tumors when implanted into the cerebellum of immuno-compromised mice. Whole brain shown (olfactory bulbs removed) with typical tumor indicated by arrow.

Post-irradiation populations contain large numbers of cells undergoing necrosis and apoptosis. Although a pure population of long-term survivors can be obtained simply by waiting for other cells to die, the time required means surviving cells proliferate extensively. A culture based model system was developed to circumvent this issue and study surviving cells as early as possible (Figure 1A).

Firstly, established medulloblastoma cell lines (Daoy and UW228) and early passage cultures of resected medulloblastoma tissue (R001M and R026M) were irradiated to generate dose response curves. This was done with the Clonogenic Colony Forming Assay (CCFA) when cells had low motility (Figure 1B), and in other cases by total viability assay (Figure 1C). CCFAs give more complete data than viability based assays, because cells must be both alive and proliferative to be counted. Differences in results from the two types of assay were observed (for early passage cultures in particular), reflecting the presence of non-proliferative cells that were damaged by radiation but that had not yet died in viability assay data. Both assays are informative for comparing the overall health of cell populations before and after radiation exposure. Our aim was to determine a dose at which between 1% and 5% of treated cells would remain viable and proliferative, such that the treatment was harsh but would provide enough survivor cells for meaningful analysis. After receiving 10 Gy, Daoy, UW228 and R001M gave 1.3%, 3.8% and 3.2% of cells (respectively) that were able to survive and form colonies (Figure 1B; R026M cells were unsuitable for CCFA).

The time course of phosphatidylserine (PS) externalization, an early step in apoptosis initiation [29], was profiled in Daoy and UW228 lines after 10 Gy irradiation, the dose chosen for resistant cell isolation (Daoy data shown Figure 1D, UW228 results similar). While high levels of external PS were readily detected after 1 day, peak levels were not reached until two days and were still significant at three days post-irradiation. For resistant cell isolation, 10 to 20 million cells were irradiated and then incubated for three days (i.e. until after the apoptotic peak). Surviving cells were then isolated by eliminating both dead (Propidium Iodide (PI) positive) cells and “doomed to die” (Annexin V positive) cells by Fluorescence-Activated Cell Sorting (FACS). Non-irradiated cells were sorted in parallel to provide a comparable control population, in which spontaneous apoptotic cells (notable in Figure 1D) were eliminated.

### Radiation tolerant medulloblastoma cells display stem behavior and are tumorigenic

Daoy and UW228 cells surviving 10 Gy radiation were able to proliferate long term in tumorsphere cultures (Figure 1E). Daoy tumorsphere cells descended from radiation survivors were assessed in an intracranial xenograft model and found to be tumorigenic *in vivo*. This confirmed that the isolated post-irradiation population included cells with cancer stem cell properties (the ability to initiate and recapitulate a tumor; Figure 1F).

### Certain stem-associated genes are elevated in medulloblastoma cells surviving high dose radiation

**Figure 2:**
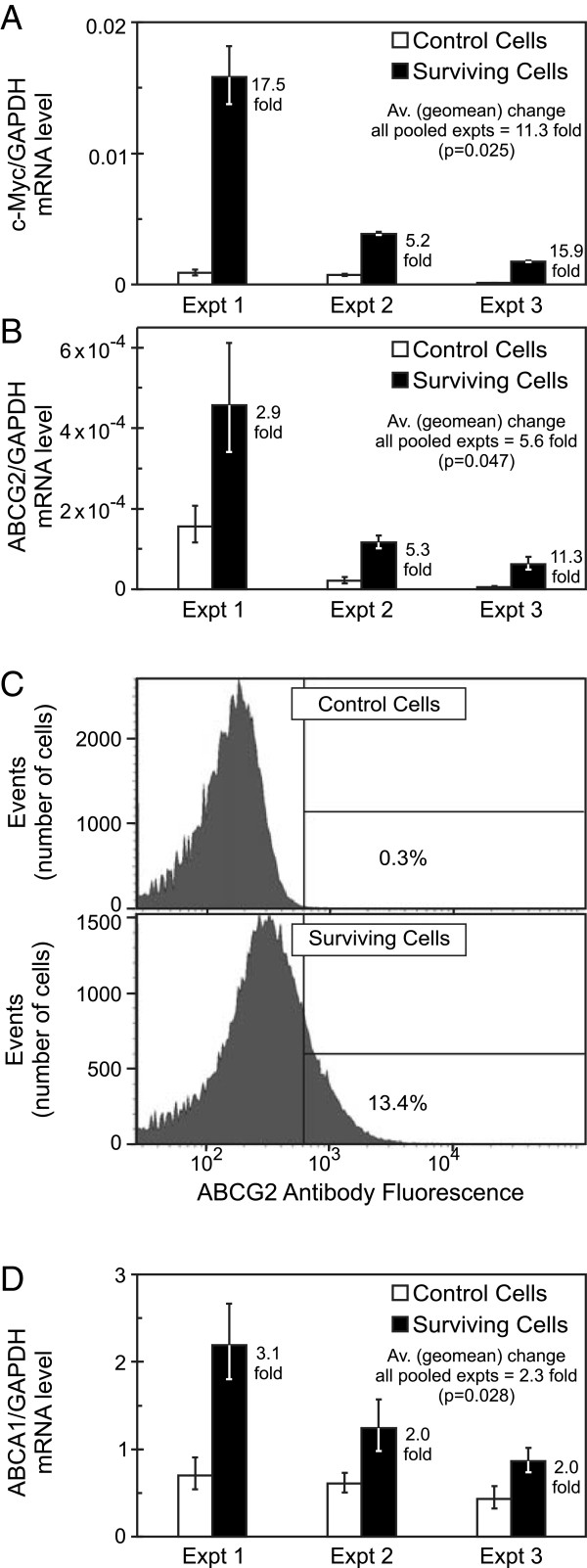
**UW228 medulloblastoma cells surviving 10 Gy radiation show elevated expression of c-Myc and several ABC transporters.** Pair-matched live non-apoptotic cell populations, from 0 Gy (control) and 10 Gy treated (surviving) cells in multiple independent experiments, were isolated using FACS. Analysis by qRT-PCR showed **(A)***c-Myc*, **(B)***ABCG2* and **(D)***ABCA1* are elevated in surviving cells. Bars show relative gene expression derived from the mean ΔCt of quadruplicate multiplex assays. To clearly display variation between experiments (both in terms of magnitudes of expression and the difference between control and resistant cells), independent experiments are shown individually. The fold-change value for each pair-matched experiment is also indicated (being the relative expression for surviving cells divided by that for control cells, or 2^-ΔΔCt^). Error bars (asymmetric on gene expression scale) represent symmetric +/− 1 SD in the Ct space. When surviving cells are compared with control cells over all pooled biological experiments, p-values are <0.05 for all three genes. **(C)***ABCG2* was also elevated at the protein level when assessed by flow cytometry.

**Figure 3:**
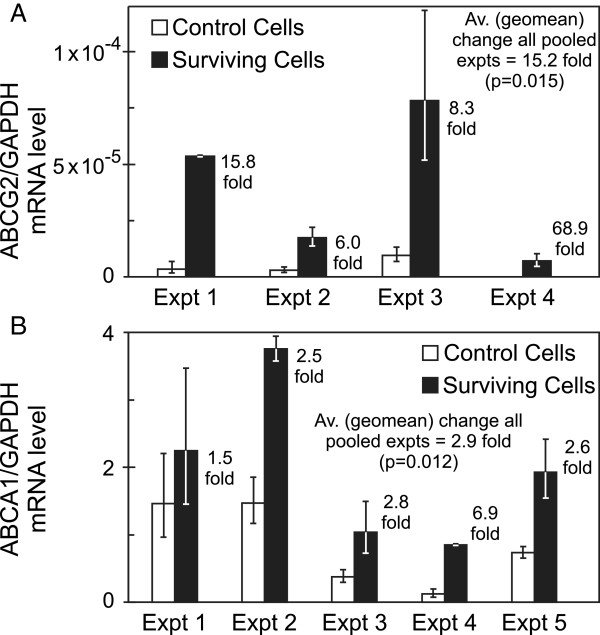
**Daoy Medulloblastoma cells surviving 10 Gy radiation show elevated expression of several ABC transporters.** Pair-matched live non-apoptotic cell populations, from 0 Gy (control) and 10 Gy treated (surviving) cells in multiple independent experiments, were isolated using FACS. Analysis by qRT-PCR shows **(A)***ABCG2* and **(B)***ABCA1* are elevated in surviving cells. Bars show relative gene expression derived from the mean ΔCt of quadruplicate multiplex assays. Independent experiments are shown individually, to clearly display variation between experiments (both in terms of magnitudes of expression and the difference between control and resistant cells). The fold-change value for each pair-matched experiment is also indicated (being the relative expression for surviving cells divided by that for control cells, or 2^-ΔΔCt^). Error bars (asymmetric on gene expression scale) represent symmetric +/− 1 SD in the Ct space. When surviving cells are compared with control cells over all pooled biological experiments, p-values are <0.05 for both genes.

A candidate gene approach was then undertaken to see if genes implicated in stem behavior were differently expressed in Daoy and UW228 radiation survivors. Quantitative Real Time PCR (qRT-PCR) was performed on the “Annexin V negative, PI negative” 0 Gy and post-10 Gy populations. At least three independent FACS isolation experiments were undertaken for each line. We did not observe any significant repeatable difference in mRNA expression with *GLI1*, *PTCH1*, *HES1*, *MSI1*, *CD133*, *nestin (NES)*, *GFAP*, *POU5F1* (*OCT4*), *SOX2*, *KLF4*, *CD15*, *CD44*, *BMI1*, *NANOG*, *survivin* (*BIRC5*), *XIAP* or *EPAS1* (*HIF2A*) in Daoy or UW228 populations (data not shown). However, two genes were of interest. For UW228 (but not Daoy) *c-Myc* (*MYC*) was consistently elevated in the radiation survivors compared with the parent population (Figure 2A) and a dramatic change in the ABC transporter *ABCG2* was repeatedly observed for both lines (Figures 2B and 3A).

Flow cytometry showed ABCG2 is also elevated in radiation survivors at the protein level (Figure 2C). Our focus then turned to this and the three related genes that play major roles in chemotherapy resistance. An additional member, *ABCA1*, was investigated as its elevation after irradiation has been reported in glioma [30]. The ABCA1 protein transports lipids and has important roles in cholesterol homeostasis and macrophage engulfment [31]. Significant mRNA changes were not observed for *ABCB1*, *ABCC1* or *ABCC2* in Daoy or UW228 radiation survivors; however the increase in *ABCA1*, as for *ABCG2*, was striking in both lines (Figures 2D and 3B).

**Figure 4:**
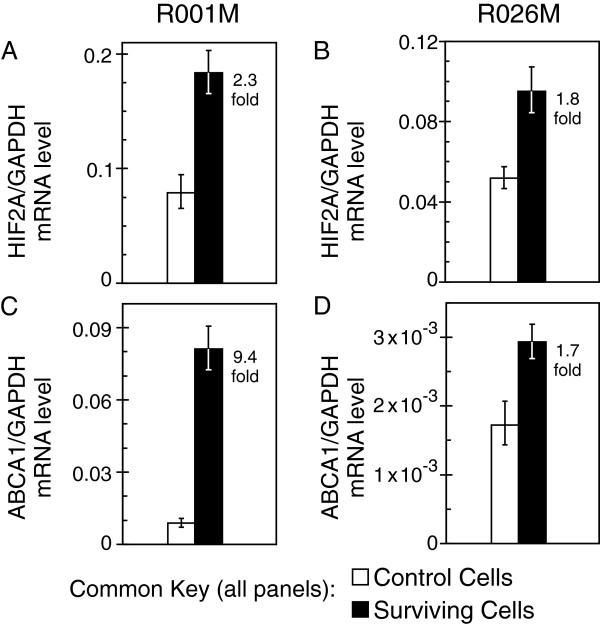
**Early passage medulloblastoma cells that survive 10 Gy radiation show elevated expression of *HIF2A *and *ABCA1*.** Live non-apoptotic cell populations of 0 Gy (control) and 10 Gy treated (surviving) cells for two newly derived human patient cultures, R001M (**A** and **C**) and R026M (**B** and **D**), were isolated by FACS. Analysis by qRT-PCR shows *HIF2A* (**A** and **B**) and *ABCA1* (**C** and **D**) are elevated in surviving cells from both patients. Bars show relative gene expression derived from the mean ΔCt of quadruplicate multiplex assays. The fold-change value for each pair-matched experiment is also indicated (being the relative expression for surviving cells divided by that for control cells, or 2^-ΔΔCt^). Error bars (asymmetric on gene expression scale) represent symmetric +/− 1 SD in the Ct space.

Early passage cultures from patient tumors (R001M and R026M) were then examined using the same FACS-based radiation survivor isolation model. Elevation of *ABCG2* and *c-Myc* was not observed in radiation tolerant cells isolated from these cultures (data not shown). However, *HIF2A* mRNA was elevated in radiation survivors from both early passage cultures, though this did not occur in Daoy or UW228 (Figure 4A and 4B). Most notable was the dramatic change in *ABCA1* levels in radiation survivors, compared with control populations, that was universally observed in all cells tested including the early passage cultures (Figure 4C and 4D).

### An alternate model involving long-term fractionated irradiation of UW228 corroborates findings of elevated *ABCA1* and *ABCG2* in surviving cells

**Figure 5:**
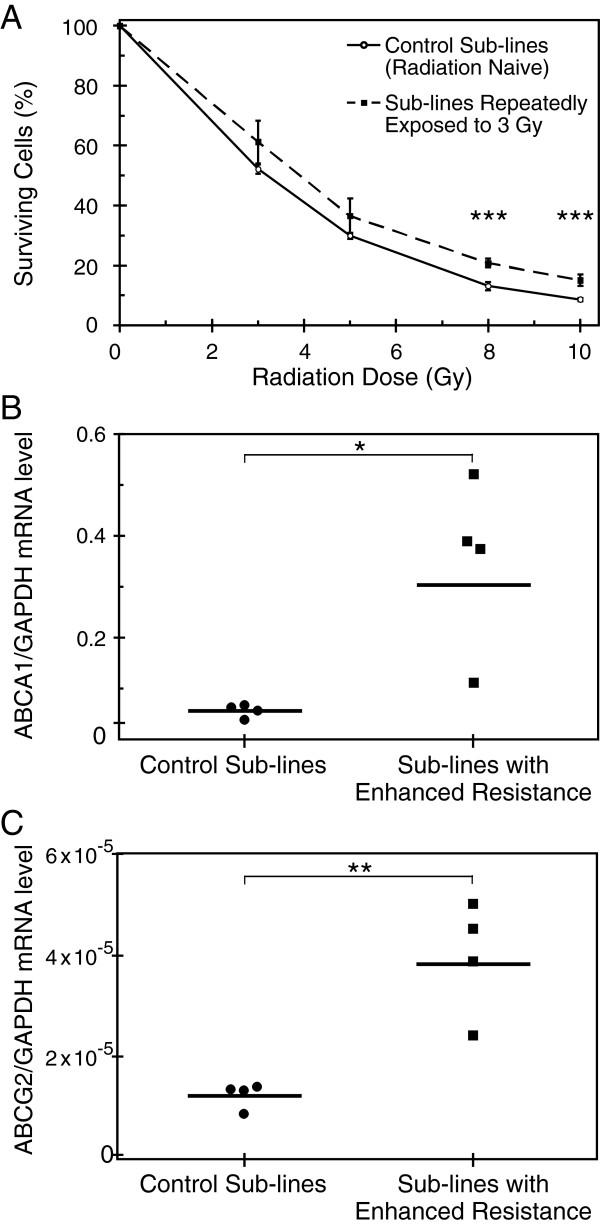
**UW228 cells show increased radiation resistance and increased ABC transporter expression after long-term weekly radiation exposure. (A)** Enhanced radiation resistance of cells exposed repeatedly to 3 Gy, by resazurin based total viability assay. Cells were assessed for resistance 2.5 weeks after receiving their final treatment dose. Mean survival of 4 long-term independently cultured sub-lines for each treatment shown. Error bars +/− 1 SD, “***” indicates p-value < 0.001. **(B)** Elevated expression of *ABCA1* and **(C)***ABCG2*, in the independent sub-lines, by qRT-PCR. Points represent relative gene expression for each line derived from the mean ΔCt of quadruplicate multiplex assays. Horizontal bars show the geometric mean for all sub-lines (“*” indicates p-value < 0.05, “**” indicates p-value < 0.01).

ABC transporter expression differences were also confirmed using an alternate strategy for studying radiation tolerant cells. Here eight sub-lines were seeded from the parent UW228 cells and four were repeatedly exposed to a moderate radiation dose (3 Gy) once per week. The other four sub-lines were passaged in parallel as controls, without radiation exposure. After twelve weeks the irradiated sub-lines displayed significantly greater resistance to radiation than the control sub-lines at high doses (8 and 10 Gy, Figure 5A). Both *ABCA1* and *ABCG2* were expressed at higher levels in sub-lines displaying increased resistance (Figure 5B and 5C).

We used FACS to isolate ABCA1 and ABCG2 expressing and non-expressing sub-populations from radiation naive UW228 and Daoy cultures, to examine whether expression of ABC transporters prior to irradiation is a marker for cells that are more likely to display radiation tolerance. Interestingly, the percentage of cells expressing either gene was very low in both lines (0.3% for ABCG2 (Figure 2C) and 1.5% for ABCA1), and positive cells did not show increased resistance over negative cells by CCFA (data not shown). Thus there may be differences in the way these genes are controlled in resistant cells in response to radiation, rather than being intrinsic markers prior to exposure.

### Verapamil mediated sensitization suggests ABC transporters may have a functional role in radiation protection

To test the hypothesis that ABC transporter proteins may provide a functional advantage to cells surviving radiation damage, we investigated a range of available ABC transporter inhibitors to see if these altered the sensitivity of Daoy or UW228 cells.

**Figure 6:**
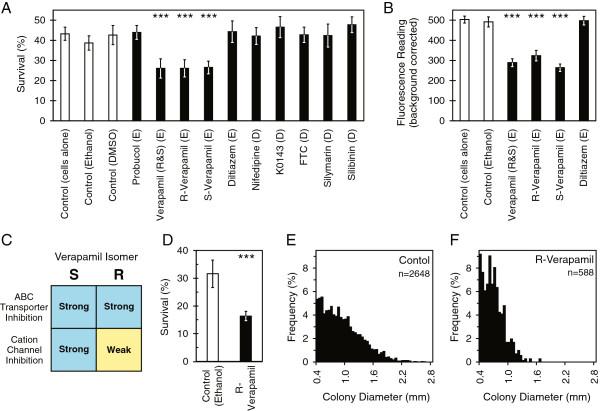
**Functional screening experiments show verapamil increases the sensitivity of Daoy medulloblastoma cells to radiation, and stunts population expansion. (A)** Investigation of inhibitors of ABC transport (and controls) and their effect on radiation response (dose = 5 Gy), using resazurin based cell viability assay. Racemic verapamil sensitized Daoy cells to radiation, as did both component isomers when used individually. “E” indicates drug carrier = ethanol, “D” indicates carrier = DMSO. Survival score was obtained by dividing mean fluorescence after drug exposure and irradiation by that of wells experiencing the drug without irradiation. Error bars +/− 1 SD. “***” indicates p-value < 0.001 when treatments are compared with carrier control. **(B)** Mean resazurin fluorescence readings for non-irradiated wells of selected treatments in Panel **A**, showing verapamil isomers lead to fewer live cells at the end of the 5 day assay period, while the calcium channel inhibitor diltiazem has no effect. Error bars +/− 1 SD. **(C)** Summary of current state of knowledge regarding the relative biological potencies of S- and R-verapamil. **(D)** R-verapamil is also a radiation sensitizer of Daoy cells in the clonogenic colony formation assay (dose = 5 Gy, 5 flasks per treatment). Error bars +/− 1 SD. **(E)** Size distribution histogram of colonies derived from non-irradiated control and **(F)** non-irradiated R-verapamil treated cells from experiment in panel **D**. Mean diameter for each colony calculated using digital image analysis.

The potential role of ABCA1 in radiation protection, of particular interest due to the universal nature of its elevation in radiation survivor cells from all patients investigated, was explored using probucol, which strongly represses ABCA1 function [32]. No effect on radiation sensitivity was observed with this compound. Likewise, specific inhibitors of ABCG2 (FTC and Ko143) failed to elicit a response. However verapamil (which inhibits multiple family members rather than just a single ABC transporter), caused a marked sensitization of Daoy cells to radiation by both total viability assay (Figure 6A) and CCFA (Figure 6D).

Verapamil is a drug with complex effects. In addition to inhibiting ABC transporters, it also blocks L-type calcium channels [11,33]. Exposing cells to other L-type calcium channel blockers (diltiazem and nifedipine) had no effect on radiation sensitivity, showing the sensitization to radiation is unlikely to be due to calcium channel effects (Figure 6A). Optical isomers of verapamil have different potencies as inhibitors, making them a useful research tool (Figure 6C). The “S” isomer possesses calcium channel blocking activity that is an order of magnitude more potent than that of the “R” isomer (dexverapamil), while both are strong ABC transport inhibitors [34-36]. Both isomers were similarly effective at sensitizing Daoy cells to radiation, further suggesting the radiation sensitizing effect is independent of calcium channel inhibition (Figure 6A).

### Transient exposure to verapamil in vitro reduces the tumorigenicity of medulloblastoma cells in vivo

**Figure 7:**
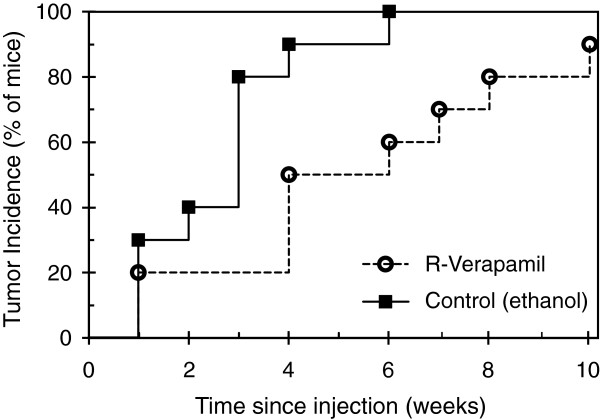
**Short exposure of Daoy medulloblastoma cells to R-verapamil *in vitro *slows xenograft tumor formation *in vivo*.** Staircase plot shows tumor incidence over time for cells exposed to R-verapamil (or carrier alone) for five days prior to subcutaneous flank injection, with n = 10 mice per treatment. Curves are significantly different by the log-rank (Mantel-Cox) test, p-value < 0.01.

Verapamil was observed to have an innate anti-proliferative effect on medulloblastoma cultures, limiting the expansion of cell populations over time (Figure 6B) and causing a distinct downward shift in the size distribution of clonogenic colonies (Figure 6F compared with Figure 6E). In light of this an *in vivo* flank xenograft model was used to determine if it affected the behavior of stem-like (tumor initiating) cells within cultures. Daoy cells were exposed to R-verapamil (or carrier) *in vitro* for 5 days and then equal numbers of live cells were implanted into mice without further treatment. This short exposure impeded Daoy tumorigenicity in the flank xenograft model. Although almost all mice had palpable tumors by 10 weeks, the majority of control xenografts were detectable within three weeks while cells treated with R-verapamil tended to display delayed tumor formation (Figure 7). Daoy cells were used in preference to UW228 cells for these experiments as the latter only gave tumors at low frequency in preliminary xenograft trials.

### Reserpine mediated sensitization also suggests a functional role for ABC transporters in radiation protection

**Figure 8:**
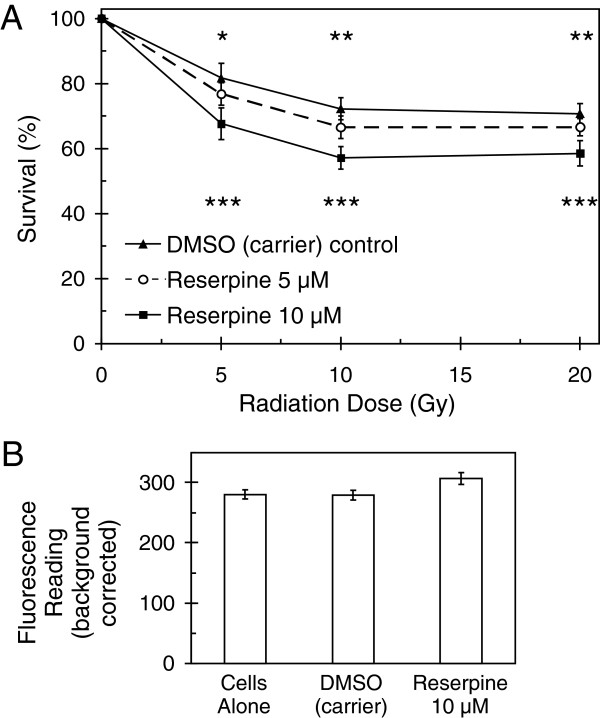
**The multi-ABC transporter inhibitor reserpine increases the sensitivity of R001M early passage medulloblastoma cells to radiation. (A)** Cells exposed to reserpine show reduced survival after high dose radiation exposure, by resazurin based total viability assay (error bars +/− 1 SD, n = 10). Asterisks above data points indicate p-values for 5 μM reserpine compared with control, asterisks below indicate significance for 10 μM (“*” p-value < 0.05, “**” < 0.01, “***” < 0.001). **(B)** Reserpine had no detectable toxicity at the highest dose used, as shown by resazurin fluorescence readings with and without drug in the absence of radiation (error bars +/− 1 SD).

When inhibitor screening was extended to the early passage cultures, sensitization to radiation was observed with reserpine, a plant derived indole alkaloid that inhibits multiple ABC transporters [11,37]. Although reserpine did not alter the dose response curves of the Daoy, UW228 or R026M cultures, it was found to increase the sensitivity of R001M cells to radiation in a dose dependent manner (Figure 8A). Conversely, the highly reproducible effect of verapamil on Daoy cells did not occur in the other lines tested (data not shown), showing that cells derived from different patients can have distinct molecular weaknesses. Reserpine was not toxic to R001M cells at the concentrations used (Figure 8B).

### ABC transporters are expressed in medulloblastoma in variable patterns and some family members are associated with particular molecular subtypes

**Figure 9:**
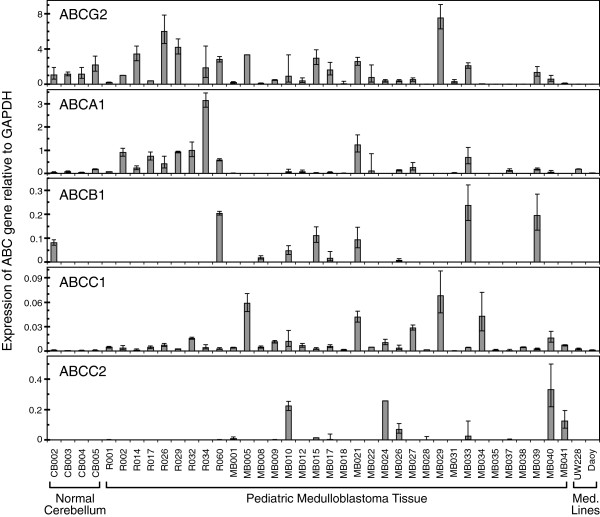
**ABC transporter levels in fresh human pediatric medulloblastoma tissue compared with medulloblastoma cell lines and normal pediatric cerebellar control tissue.** Expression of five key members of the ABC Transporter family, relative to GAPDH expression, as shown by qRT-PCR. Values were derived from the mean ΔCt of triplicate multiplex assays. Error bars (asymmetric on gene expression scale) represent symmetric +/− 1 SD in the Ct space.

Since certain ABC transporters: 1) mark cells with stem-like behavior in various systems, 2) have roles in chemotherapy resistance and 3) may be involved in radiation protection, we surveyed expression of key ABC transporters in freshly resected pediatric medulloblastoma tissue. Investigation of 34 cases by qRT-PCR showed *ABCG2* and *ABCA1* were often expressed (91% and 82% of patients respectively), while *ABCC1* was expressed universally. In contrast, *ABCB1* and *ABCC2* were only detected occasionally (44% and 29% of patients respectively). Patterns of ABC transporter expression were highly variable from patient to patient, and levels for one or more ABC transporter genes were frequently elevated compared with normal pediatric cerebellum (Figure 9).

As molecular sub-typing data was unavailable for the majority of our patient cohort, we turned to a large and well annotated public medulloblastoma dataset for further investigations [38]. As in our dataset, ABC transporter genes tended to be expressed at varying levels for these 62 cases (results shown in Additional files 1, 2, 3). Notable patterns were discovered in the expression of several ABC transporters when tumors were clustered by subtype. Of the five genes focused on in our study, *ABCA1* was of note, as high expression was significantly associated with group A (WNT associated) tumors and low expression with groups B (SHH associated) and E. *ABCB1* tended to be more highly expressed in group B, C and D than in the other two groups (refer Additional file 1; Note that groups C and D in this dataset are equivalent to “group 4” in the current consensus nomenclature [39]). The most striking associations however were with other family members. Our initial analysis identified a number of ABC transporters that show significant subtype-related expression trends, with the highest significance discriminators being *ABCA8, ABCB4, ABCC8 and ABCD2* (refer Additional files 2 and 3).

**Figure 10:**
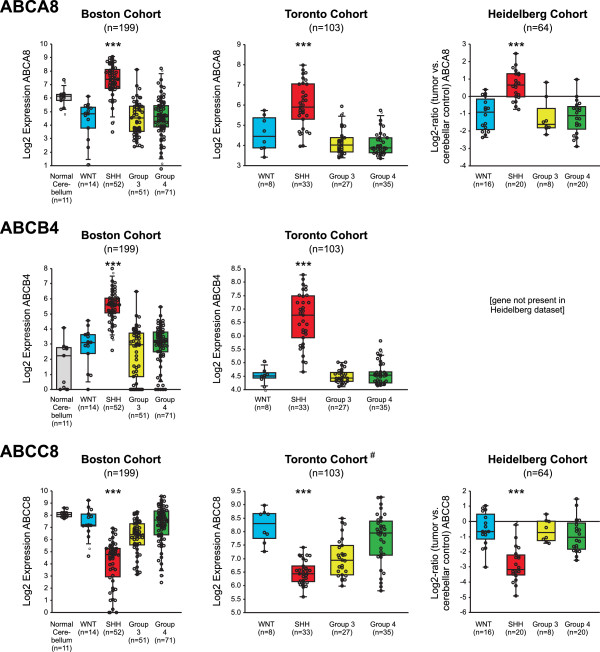
***ABCA8*, *ABCB4 *and *ABCC8 *show distinctive SHH subtype associated expression patterns, relative to other medulloblastoma subgroups and normal cerebellum.** Expression data from the three independent validation cohorts (n = 366 patients total) shown as box and whisker plots (quartiles and median indicated by box outline and centreline respectively). Asterisks indicate significance of difference in expression between subgroup of interest and the remaining subgroups (“***” = p-value < 0.001). In addition, comparisons between the subgroup of interest and normal cerebellum (available for the Boston cohort) were significantly different with a p-value of 0.01 or better for all genes shown. [Note that in the Toronto experiments some probe sequence overlap was present between *ABCC8* and enzyme component gene *SDHC*, for the plot indicated with “#”. Analysis of independent *SDHC* probes in the other analysis sets showed that the SHH subgroup effect seen for the Toronto cohort is likely driven largely or entirely by *ABCC8*. There were no confounding issues with the probes for *ABCC8* used for the Boston or Heidelberg cohorts].

These findings were then validated using data from three large independent cohorts [40-43] that included 366 medulloblastoma cases. Expression “heat maps” for all available ABC transporter family members are shown in Additional file 4. Previously observed trends were corroborated, including high expression of *ABCA1* in the WNT subgroup (refer Additional file 5). Data confirming the association of high *ABCA8* and *ABCB4* and low *ABCC8* expression with the SHH subgroup (relative to other subgroups and normal cerebellum) is shown in Figure 10.

## Discussion

While incremental improvements to surgical, chemo-therapeutic and radiological procedures continue to increase survival for pediatric brain tumor patients, death rates and quality of life issues for those who remain in remission are significant. The key to overcoming limitations of existing treatments is to increase our understanding of tumor cell biology at the level of individual cells.

Towards this goal, we used a functional approach to investigate medulloblastoma cells displaying high radiation tolerance. FACS was employed to remove dead and apoptotic cells from irradiated cultures, so that surviving cells could be studied before extensive cell division took place. This “rapid isolation approach” allowed study of the tolerant cells or their early descendants, rather than distant progeny. The “survivor cells” displayed tumorigenic potential *in vivo*, showing that they were indeed “healthy” and retained stem-like behavior. The finding of elevated levels of *ABCG2* in such cells from a subset of human patients, and of *ABCA1* in all patient cultures, was of particular interest given the important roles ABC transporters play in chemo-resistance and stem cell biology. *ABCG2* has also been implicated in cancer susceptibility, as a polymorphism in the human gene is associated with a change in incidence for several malignancies [44]. In this study we chose to focus on a key subset of ABC transporters known to be strong drivers of drug resistance in human patients, or that play a known role in stem cell or brain tumor biology. It is anticipated that other members of this extensive gene family may also display increased expression in medulloblastoma cells after radiation treatment.

Elevated levels of ABC transporters may be observed in radiation tolerant cells for three reasons: they could reflect a generalized stress response, they could provide a functional advantage to irradiated cells, or their expression might be correlated with other genes that are functionally involved in resistance. The fact that both *ABCA1* and *ABCG2* were elevated in an independent model of radiation resistance in UW228 cells led us to further explore the role of the ABC transporter family. Findings with *c-Myc* and *HIF2A* in various patient cultures are also of interest, and are a focus of ongoing investigation.

*ABCA1* and *ABCG2* proteins were only expressed on a small proportion of cells within radiation naive medulloblastoma lines. Subsequent analysis showed their expression did not specifically mark cells that would better survive radiation exposure. This suggests cells with greater intrinsic resistance may exist in a “primed” state and have the ability to express such genes after radiation is experienced.

Difficulties were anticipated in using RNA-based knock-down technology to explore the possible functional relevance of expression changes, due to the vast number of ABC transporter genes in the human genome and their tendency for overlapping roles, and the fact that we wished to investigate early passage primary culture cells (which are challenging to transfect efficiently). Instead, we took advantage of the wide range of readily available compounds known to inhibit single or diverse ABC transporter proteins. Use of these led to our discovery that ABC transporters not only contribute to chemotherapy resistance, but also play a functional role in radiation protection. This finding is based on studies with ABC transporter inhibitors of two distinct molecular classes. Although the multi-ABC transporter blockers verapamil and reserpine enhanced radiation sensitivity, compounds specifically inhibiting just ABCG2 or ABCA1 had no detectable effect. This suggests overlapping function of genes, with multiple transporters having functions that together contribute to radiation resistance. Thus it appears combinations of transporters need to be blocked to prevent radio-protective effects. The fact that reserpine and verapamil sensitized cultures from different patients may be a reflection of the varied ABC transporter expression patterns observed between different medulloblastoma cases, in both our own and publicly available gene expression datasets.

Results obtained with verapamil and reserpine need to be interpreted with care, since their channel inhibition effects extend further than the ABC transporter family. Verapamil also possesses anti-L-type calcium channel activity and influences potassium transport. Our finding that the R-verapamil optical isomer mediates radiation sensitization at a level indistinguishable from that of S-verapamil, even though the former only possesses weak calcium/potassium channel activity, is important given both are similarly potent in blocking ABC transporters [34-36,45,46]. This suggests the radiation protection mechanism is ABC transporter driven, although it is possible that a residual level of anti-cation channel activity could saturate a sensitive system. Our second line of evidence suggests this is not the case, as deliberate addition of other inhibitors of L-type calcium/potassium activity did not have any observable effect on radiation sensitivity. While such experiments implicate ABC transporters, this does not preclude verapamil having effects on other undiscovered cellular targets that may also protect cells from radiation. However, observation of a similar radiation sensitizing effect with the independent ABC transporter inhibitor reserpine further strengthens the case for ABC transporter involvement.

Reserpine is used extensively in research due to its potent inhibition of ABC transporters [11,37]. While verapamil and reserpine overlap in their inhibition of ABCB1, their effects on the majority of other individual family members remain unstudied [11]. In addition to ABC transporters, reserpine also inhibits the vesicular monoamine transporter (VMAT), which is involved in neurotransmitter movement in the CNS [47]. Although less likely in light of the verapamil result, it remains possible that the dose dependent sensitization to radiation observed with reserpine was due to neurotransmitter rather than ABC transporter effects. If this is the case, it would raise the intriguing possibility that reserpine may influence radiation responses by directly manipulating the stem-like properties of cells, since inhibitors of neurotransmitter pathways are known to reduce neural stem cell populations [48].

The anti-proliferative effect of verapamil observed in medulloblastoma lines supports earlier reports of this phenomenon in various neoplastic cells [49-53]. Though initially presumed to be due to calcium effects, there is now evidence that the anti-cancer properties of verapamil involve non-calcium dependent mechanisms [52,54]. Verapamil is also anti-tumorigenic when given to live animals harboring meningioma xenografts [55]; however our study is the first to show transient exposure to verapamil *in vitro* has long lasting effects when tumorigenicity is subsequently assessed *in vivo*. This innate anti-tumorigenic effect may provide a therapeutic bonus (in addition to chemo- and radiotherapy sensitization), if new generation drugs with structures similar to verapamil are used in the treatment of future patients.

Increased expression of ABC transporters, particularly *ABCB1*, has been reported after irradiation in several cell types [56-58]. The exact mechanism by which this is controlled remains largely unexplored, although a number of ABC transporters are known to be up-regulated in response to xenobiotics or stimuli that indicate harsh conditions. One such condition is hypoxia, which is known to influence expression via the binding of Hypoxia Inducible Factor (HIF) complexes to ABC transporter promoters [59]. Oxidative stress is also known to stimulate ABC transporter expression, for example activation of the *ABCG2* promoter can be mediated by the transcription factor NRF2 under such conditions [60]. It is possible that this, or alternate transcription systems that detect oxidative stress or cellular damage, may be responsible for up-regulating ABC transporter family members after irradiation in medulloblastoma.

The fact that observed gene expression changes upon irradiation in R001 and R026 cells were more similar to each other than to other investigated cells led us to investigate the subtype classification for these patients. Both were found to represent “Group 3” tumors by 22 gene signature nanoString nCounter analysis ([61] and data not shown). Both UW228 and Daoy have previously been analyzed by other researchers [62], and found to be of the SHH subtype. This may in part explain the variability observed between the responses, particularly since elevation of *ABCG2* in response to radiation was observed for UW228 and Daoy but not for R001 or R026. The transcription of certain ABC transporter genes, including *ABCG2*, is known to be directly influenced by activation of the Hedgehog signaling pathway [63-65].

While ABC transporter related responses to drugs have been investigated by numerous groups, a functional role for ABC transporters in radiation protection remained largely unexplored prior to our studies. However, a hypothetical radio-protective role has previously been proposed [66], and a precedent for the transport of molecules as a mechanism of radiation protection does exist, with the non-ABC transporter RLIP76 known to play a key role in glutathione-mediated radiation protection in non-cancerous cells [67]. ABC transporters may potentially protect cells from radiation via direct transport of toxic radiation by-products from cells, by moderating glutathione balance, or by actively removing glutathione-bound conjugates. Alternatively, the effect may be more indirect, such as ABC transporters shuttling post-irradiation “messenger molecules” between compartments or cells. Such effects could in turn trigger DNA repair or modulate the apoptotic response in order to enhance cell survival. There is evidence from leukemia cells suggesting that verapamil-reversible drug resistance can be driven by glutathione based systems, rather than drug efflux [66]. Additionally, over-expression of *ABCB1* in hematopoietic cells appears to reduce apoptosis [68]. ABC transporters may contribute to radiation resistance via similar means. Elucidation of the exact mechanism awaits further study.

In light of our findings supporting a functional role for ABC transporters in radiation protection, two previous studies are of note. In 2009 a stem-like sub-population of bladder cancer cells with high ABC transporter activity was shown to be sensitized to radiation in the presence of verapamil [69]. This study did not attempt to determine if this was mediated by cation channel effects or ABC transporter blockade. Our findings show pediatric medulloblastoma behaves similarly, and that the mechanism is likely to be independent of cation effects. Further evidence supporting our finding of ABC transporters as mediators of radiation resistance comes from a study of breast cancer patients. A single nucleotide polymorphism screen has previously shown that patients harboring an amino acid change in *ABCA1* tend to suffer radiation induced dermatitis during therapy [70], implying *ABCA1* is a contributor to cellular radio-protection, at least in some cell types. While the exact combination of members in this large family that play a role in the radiation tolerance phenotype is yet to be determined, our finding that certain multi-ABC transporter inhibitors can sensitize cells to radiation whilst single transporter specific agents lack an effect, indicates that a number of ABC transporter proteins are involved, probably with redundant functions.

We utilized a large publicly available dataset [38], to complement our initial qRT-PCR study and provide the first family-wide survey of ABC transporter expression in medulloblastoma. The observed variation in expression patterns contributes to our finding that ABC transporter inhibitors give differing effects on radiation tolerance depending on the patient that cell cultures are derived from.

Our analysis of publicly available microarray data showed that the expression level for several ABC transporter family genes is strongly associated with particular medulloblastoma subtypes. This was particularly evident with *ABCA8*, *ABCC8 ABCD2* and *ABCB4*. Potential correlations between ABC transporter expression and relapse/clinical progression will be explored as our cohort matures. This information was not available for the dataset used for the discovery of subtype related associations, however evidence from multiple groups has shown robust correlation between subtype and clinical outcome (in particular a relatively favorable outcome for WNT driven tumors and a poorer than average outcome for “Group 3” medulloblastoma) [39,71,72].

The fact that certain ABC transporters show distinct patterns of expression for particular subtypes, and that the proteins are membrane localized, makes them attractive candidates as novel diagnostic markers (particularly for discriminating between SHH and non-SHH pathway driven tumors). In will be interesting in future studies to see if this association also extends to other tumor types.

Whether the suite of ABC transporters that play a role in radiation protection overlap with the family members that show strong subtype dependent expression patterns is also of particular interest for future study. Further study of ABC transporters stands to provide key insights into the biology of how certain cancer cells evade radiation-induced death.

## Conclusions

Our work provides proof of principle for a novel method of enrichment for radiation tolerant medulloblastoma cells isolated early after gamma ray exposure. We further demonstrate that these tolerant cells retain tumor initiating properties. Success with an initial candidate set has provided insight into the expression of genes related to stem-like biology in cells that survive radiation exposure. The observed elevation of ABC transporter expression in these cells has implications for the order in which treatments are delivered to patients, as increased efflux pump activity may reduce the efficacy of chemotherapeutic drugs that are delivered after radiotherapy.

For the first time, functional data is presented suggesting that ABC transporter proteins contribute to cellular radiation protection mechanisms. Our *in vitro* studies suggest that multiple ABC transporter proteins likely act in concert to contribute to radiation tolerance in human medulloblastoma cells. The fact that broad spectrum inhibitors significantly sensitize cells, whilst those targeting individual ABC transporters do not, suggests an overlap in the roles of the various family members involved in this newly detected mechanism for radiation protection.

Although clinical trials of ABC transport inhibiting compounds aimed at circumventing chemotherapy resistance initially hit obstacles, promising “new generation” anti-ABC transporter drugs are now at various stages of pre-clinical and clinical assessment [9,73,74]. Our work suggests such compounds, if they inhibit multiple transporters, could potentially provide additional benefits for patients if they are delivered during radiation as well as chemotherapy treatment.

Most importantly we also show, through analysis of publically available gene expression data sets, that certain ABC transporters contribute to gene expression signatures for particular molecular subtypes of medulloblastoma, a finding which could be exploited in future prognostic tests.

## Materials and methods

### Medulloblastoma patient tissue, lines and culture conditions

Both Daoy (desmoplastic variant, from American Type Culture Collection; HTB-186) and UW228 cells were cultured in MEM alpha with 9.1% fetal bovine serum (FBS) [75,76]. Fresh medulloblastoma tissue was obtained from patients at the time of surgery. Portions were snap frozen for later RNA extraction and the remainder was finely chopped with a scalpel and cultured in DMEM/F12 with 9.1% FBS. After one week, growing explants were trypsinized and seeded for continuing adherent cell culture. Monolayer cultures from patients R001 and R026 (designated R001M and R026M) were used in experiments prior to passage 15. Tumorspheres were cultured using neurosphere conditions [77]. All culture reagents were obtained from Invitrogen. Patient material was ethically consented and used in accordance with approvals from The University of Queensland Human Research Ethics Committee (HREC) and the Queensland Children's Health Services (RCH) HREC (approval #2006000106 and #2005/077 respectively). Further ethically consented medulloblastoma and normal pediatric cerebellum samples (from autopsies after fatal trauma), were obtained from the Fred Hutchinson Cancer Research Center, Seattle, USA. Clinical details for investigated patients are given in Table 1. All human subject work conformed to the Helsinki Declaration (2008 revision).

**Table 1:** Human medulloblastoma patients for which tumor tissue was used for gene expression and/or culture experiments

**Patient**	**Pathology at diagnosis**
**ID**	
R001	Medulloblastoma, classic
R002	Medulloblastoma, classic
R014	Medulloblastoma, classic
R017	Medulloblastoma, classic
R026	Medulloblastoma, poorly differentiated classic
R029	Reported by independent pathologists as 1) Medulloblastoma and 2) unusual AT/RT-like tumor (without INI1 loss)
R032	Medulloblastoma, classic, with metastatic nodule nearby
R034	Medulloblastoma, classic
R060	Medulloblastoma, with extensive nodularity
MB001	Medulloblastoma, recurrent
MB005	Medulloblastoma, recurrent
MB008	Medulloblastoma, desmoplastic
MB009	Medulloblastoma, classic
MB010	Medulloblastoma, classic
MB012	Medulloblastoma, classic
MB015	Medulloblastoma, classic
MB017	Medulloblastoma, classic
MB018	Medulloblastoma, desmoplastic
MB021	Medulloblastoma, classic
MB022	Medulloblastoma, classic
MB024	Medulloblastoma, classic
MB026	Medulloblastoma, classic
MB027	Medulloblastoma, classic
MB028	Medulloblastoma, classic
MB029	Medulloblastoma, classic
MB031	Medulloblastoma, recurrent
MB033	Medulloblastoma, classic
MB034	Medulloblastoma, classic
MB035	Medulloblastoma, classic
MB037	Medulloblastoma, classic
MB038	Medulloblastoma, classic
MB039	Medulloblastoma, classic
MB040	Medulloblastoma, desmoplastic
MB041	Medulloblastoma, desmoplastic

### Radiation exposure

Cells were seeded and allowed to adhere for two to five hours prior to radiation delivery via a Gammacell 40 irradiator (Nordion). For survivor cell enrichment, cells were exposed to a single 10 Gy dose (except for R026M for which 60 Gy was used) and FACS performed three days after radiation exposure. Populations undergoing repeated weekly radiation exposure were rested for one week after the final dose, before gene expression analysis was undertaken. This delay was to ensure that any potential “stress effects” of the radiation treatment itself would not confound analysis of the surviving populations.

### Immunofluorescence and flow cytometry

PS externalization was detected using Annexin V conjugated to Alexa 488 or Pacific Blue (#A13201 or #A35122, Invitrogen). Dead cells were detected with PI or 7-ADD (#P21493 or #A1310, Invitrogen). Immunofluorescence was performed using standard methods after fixation with 4% paraformaldehyde. A FACS ARIA Cell Sorter was used for cell isolation and a FACS Canto II for general flow cytometry (both BD Biosciences). ABCG2 was detected with an Alexa Fluor 700 labeled antibody (#332016; BioLegend) and ABCA1 with a DyLight 488 conjugate (#NB400-105G; Novus Biologicals).

### Treatment with channel blocking compounds

Verapamil (R and S mixture), R-verapamil and S-verapamil were from Sigma-Aldrich (#V4629, #V106 and #V105 respectively). Other inhibitors were also obtained from Sigma-Aldrich, except for the ABCG2 specific blockers fumitremorgin C (FTC) and Ko143, which were from Enzo Life Sciences (#ALX-350-127-C250 and #BML-EI396-0025) [78]. Stocks were prepared in ethanol or dimethyl sulfoxide (DMSO) as appropriate, and the carrier used as a control. Compounds were used at 50 μM unless otherwise stated, with the exception of FTC, Ko143 and reserpine, which were routinely used at 10 μM. Drugs were added 1 to 2 hours before radiation treatment for inhibitor screening. Media was changed post-irradiation and fresh drug added, to circumvent potential damage to compounds from radiation itself, and fresh media/drug was again added after 24 hours.

### Assessment of cell viability and colony forming ability

Total cellular viability assays were performed in 96 well plates using the resazurin based “CellTiter-Blue” assay (#G8081, Promega). A minimum of six replicate assays were performed per treatment and cells were incubated for five to nine days post-treatment, until control wells were 90% confluent. Fluorescence was measured using a Fluorocount reader (Perkin Elmer).

Colony formation was assessed using the Clonogenic Colony Forming Assay (CCFA). Cells were treated after seeding at low density then incubated (six days for UW228, seven days for Daoy and 28 days for R001). A minimum of five 25 cm^2^ flasks were used per treatment, such that at least 2500 cells were assessed. Colonies were counted and sized from high resolution whole flask digital photographs with automated image analysis software (Analysis LS Research, Olympus). Clusters were not counted as colonies unless cells had achieved five to six population doublings (minimum mean diameter of 0.4 mm) [79].

### RNA and gene expression analysis

RNA was prepared with RNeasy columns (#74104, Qiagen). Synthesis of cDNA was carried out with Superscript III (#18080-085, Invitrogen) and qRT-PCR was performed with an ABI 7900HT system using TaqMan gene expression assay primer-probe sets (Applied Biosystems). Relative expression was determined using ΔCts, from triplicate or quadruplicate reactions, using multiplexed GAPDH as the endogenous reference.

Subgroup affiliations for tumors from patients R001 and R026 were determined using nanoString-based targeted gene expression profiling as previously described [61]. Normalization of nanoString data and subgroup prediction were performed using the R statistical environment (v 2.15), as previously described [61].

The publicly available medulloblastoma gene expression array data used for initial analysis was obtained from the National Center for Biotechnology Information (NCBI) Gene Expression Omnibus (GEO; http://www.ncbi.nlm.nih.gov/geo/; identifier GSE10327 [38]) and analyzed using the NCBI GEO2R package. Significance testing was performed conservatively using the Benjamini and Hochberg multiple-testing correction, to minimize the false discovery rate [80].

Validation data from three further cohorts (“Boston” [40], “Toronto” [41] and “Heidelberg” [42,43]), were analyzed using R2 (http://hgserver1.amc.nl/cgi-bin/r2/main.cgi). The KEGG geneset annotated as “ABC transporters” was visualized using heat maps according to subgroup affiliation and in normal tissue if applicable. One-way ANOVA and post-hoc comparisons were used to determine gene expression differences across subgroups and between subgroups, respectively. P-values below 0.05 were considered significant.

### Murine xenograft models for in vivo human tumor growth

Orthotopic cerebellar xenografts were performed using a stereotaxic platform and micro-drill to make a burr hole 1 mm lateral to the sagittal suture and 1 mm posterior to the lambdoidal suture. 100,000 cells were then injected 3 mm below the skull surface using a Hamilton syringe (delivered over three minutes and needle withdrawn slowly after a further minute). For *in vivo* tumorigenicity testing after *in vitro* verapamil exposure, 50 μM of drug was added to cells in culture and media changed and fresh drug added after 24 hours. The cells were then incubated for a further four days before trypsinization and delivery of 200,000 live cells per injection. Cells were delivered subcutaneously in the lateral mid-flank, in a 200 μl volume with 50% matrigel (#356234, BD Biosciences). Mice were examined for presence of palpable tumors twice per week thereafter. All experiments used female severe combined immunodeficiency mice. Animal studies were performed in accordance with approvals obtained from the Health Sciences Animal Ethics Committee of The University of Queensland (approval #RCH/118/09).

### Statistical analyses

Significance testing of large scale gene expression data is outlined above. For other experiments, statistical analysis was performed using GraphPad Prism5 software. For qRT-PCR data (presented as the level of expression for the gene of interest relative to GAPDH), significance was assessed using the ratio t-test (two-tailed paired t-test on log transformed data). Error bars on CCFA and viability assay graphs represent the standard deviation (SD) for the numerator (treated) and denominator (control) components of each survival calculation, combined (as percentages of the associated means) using the “square root of the sum of squares” technique. Tumor formation in mice over time was assessed using the log-rank (Mantel-Cox) test. For all other experiments, p-values were derived using two-tailed t-tests.

## Abbreviations

ABC: ATP-binding cassette; CCFA: Clonogenic colony forming assay; CNS: Central nervous system; CSC: Cancer stem-like cell; DMEM/F12: Dulbecco's modified eagle medium/nutrient mixture F-12; DMSO: Dimethyl sulfoxide; FACS: Fluorescence-activated cell sorting; FTC: Fumitremorgin C; FBS: Fetal bovine serum; PI: Propidium iodide; PS: Phosphatidylserine; qRT-PCR: Quantitative real-time PCR; SD: Standard deviation; SHH: Sonic hedgehog.

## Competing interests

The authors do not have any conflicts of interest to declare.

## Authors' contributions

WJI conceived the study, performed *in vitro* and *in vivo* experiments, interpreted the data, performed statistical analyses and wrote the manuscript. DV performed *in vitro* and *in vivo* experiments. IH, LMC, RF and participated in study design, performed culture based assays, qRT-PCR, cytometry, *in vivo* experiments and data interpretation. EBL participated in study design, performed culture based assays, and assisted with *in vivo* experiments and manuscript revision. TEH facilitated access to fresh patient tissue and clinical data. MR and MDT performed subtyping and provided data and statistical analysis for the validation cohorts. ARH participated in the study design, coordinated the project and assisted with manuscript revision. All authors read and approved the manuscript.

## Supplementary Material

Additional file 1 **Expression profiles for ABC transporters of particular interest, by molecular medulloblastoma subtype.** Expression patterns from GEO2R analysis of dataset NCBI GEO GSE10327 (62 human medulloblastoma cases, organized by subtype), for *ABCA1* and four key modulators of drug resistance in human cells (*ABCG2*, *ABCB1*, *ABCC1* and *ABCC2*). Click here for file

Additional file 2 **Differential expression analysis summary for all ABC transporter family genes present in dataset NCBI GEO GSE10327, by molecular subtype.** Statistical significance data matrix for all subtype comparisons with all represented ABC gene family members (in 62 medulloblastoma cases), by GEO2R analysis. Relationships with p-values < 0.001 are highlighted. Click here for file

Additional file 3 **Expression profiles for four ABC transporters that show highly significant medulloblastoma subtype associations.** Expression patterns from GEO2R analysis of dataset NCBI GEO GSE10327 (62 human medulloblastoma cases), for ABC transporters found to have combinatorial subtype discrimination p-values < 0.000001 (*ABCA8*, *ABCC8*, *ABCD2* and *ABCB4*). Click here for file

Additional file 4 **Medulloblastoma Gene Expression Heat Maps for ABC Transporter Family Members. Expression data for available ABC Transporter genes in the Boston and Toronto sets, shown as heat maps (over-expression = red, under-expression = green).** Patients are ordered by molecular subtype and clustered by ABC expression pattern. Click here for file

Additional file 5 ***ABCA1* is highly expressed in WNT driven medulloblastoma, relative to other subgroups and normal cerebellum.***ABCA1* expression data from the three independent validation cohorts (n = 366 patients total), shown as box and whisker plots (quartiles and median indicated by box outline and centreline respectively). Asterisks indicate significance of difference in expression between the WNT tumors and the remaining subgroups (“***” = p-value <0.001). In addition, comparison between the WNT subgroup and normal cerebellum (available for the Boston cohort) showed a significant difference, with a p-value < 0.001. Click here for file
