# Supplementary material for: ABC transporter activity linked to radiation resistance and molecular subtype in pediatric medulloblastoma
Source: Exp Hematol Oncol. 2013 Oct 4;2:26. doi: 10.1186/2162-3619-2-26 (PMC3851566; doi:10.1186/2162-3619-2-26)

Ingram *et al.* Additional File 1:

Expression profiles for ABC transporters of particular interest, by molecular medulloblastoma subtype

Probeset expression profiles in public dataset NCBI GEO GSE10327 (62 Medulloblastoma Cases by Subtype). X-axis scale = Affymetrix data signal strength value. Note probesets for multiple transcripts are present for some genes.

Group names are as in Kool *et al*., 2008. (Group A = WNT associated, Group B = SHH associated, while Group C + Group D = Group 4 and Group E = Group 3 in consensus nomenclature of Taylor et al. 2012)

ABCA1

Probeset 203504_s_at discriminates group A from BCDE with an Adj. p-value **<0.0001** and BE from ACD with an Adj. p-value **<0.0001**

Probeset 203505_at discriminates group A from BCDE with an Adj. p-value <0.0001 and BE from ACD with an Adj. p-value **<0.001**

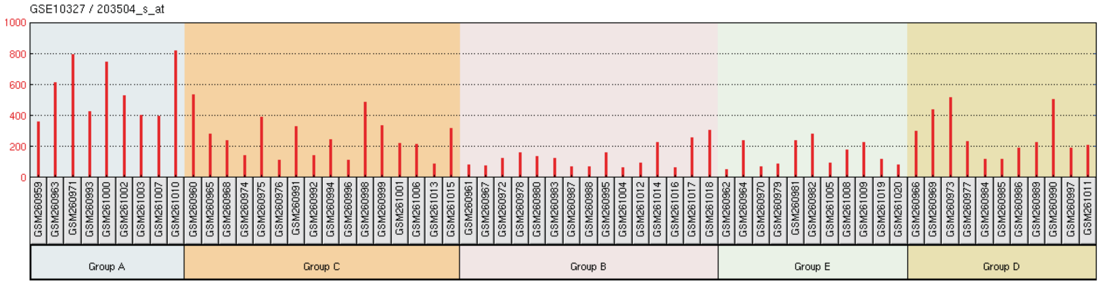

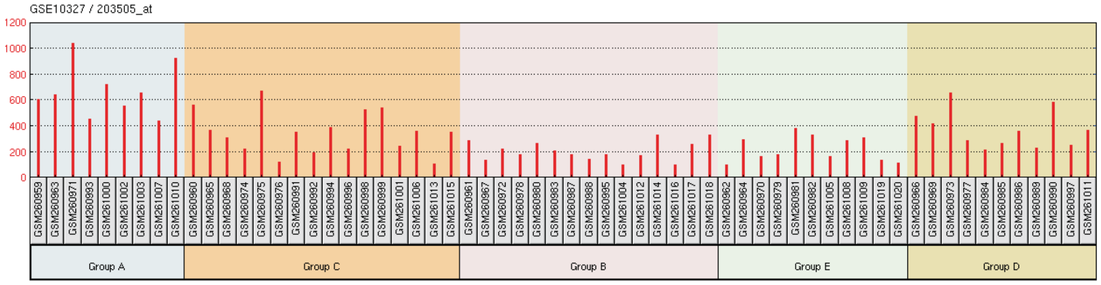

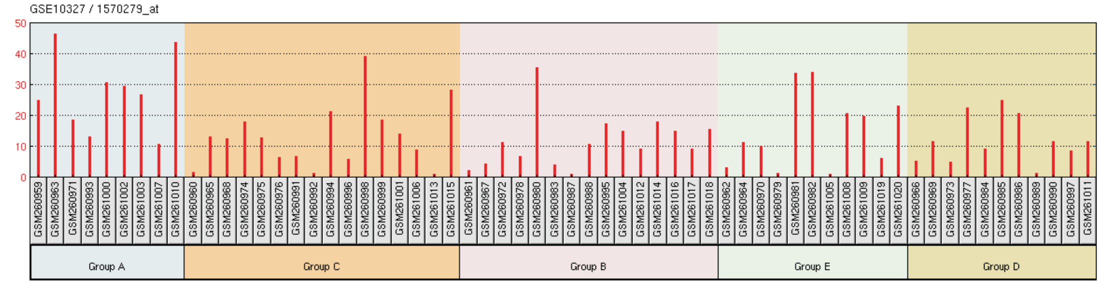

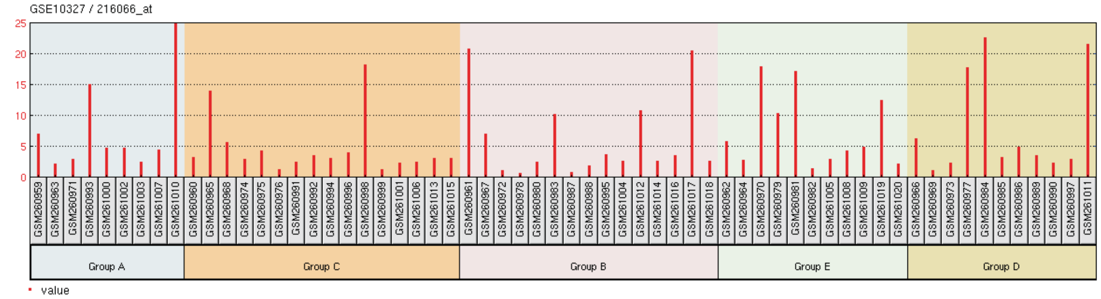

ABCG2

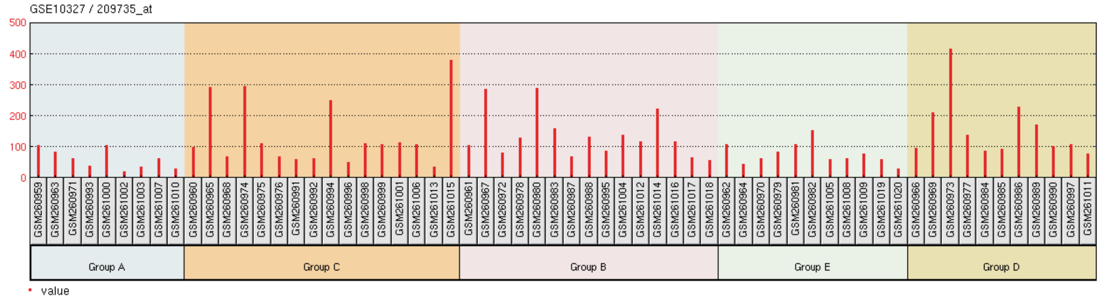

Ingram *et al.* Additional File 1 continued...

ABCB1

Probeset 209993_at discriminates group A from BCDE with an Adj. p-value **<0.001** and AE from BCD with an Adj. p-value **<0.0001**

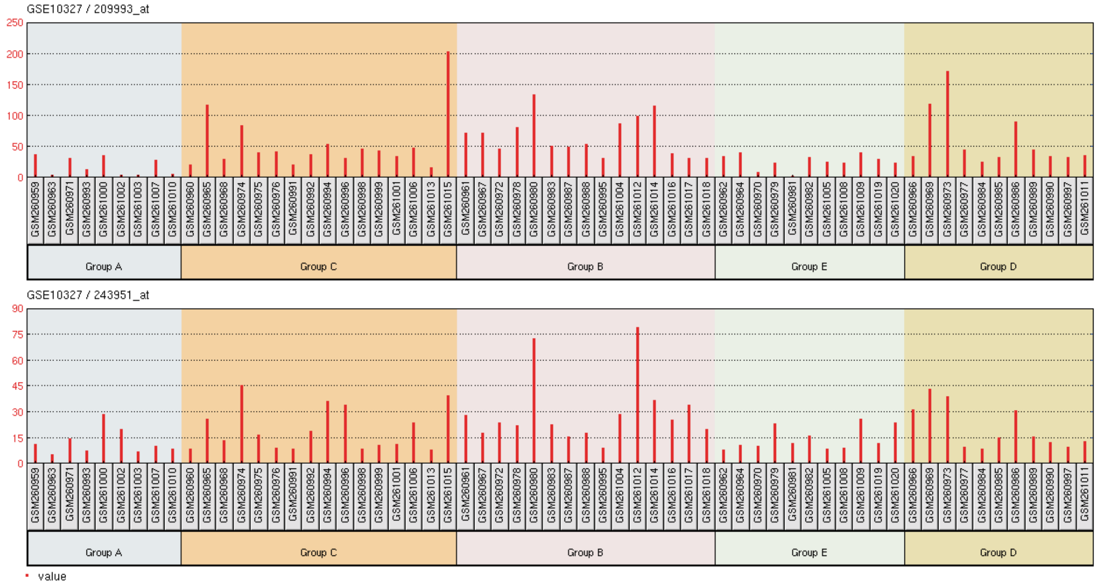

ABCC1

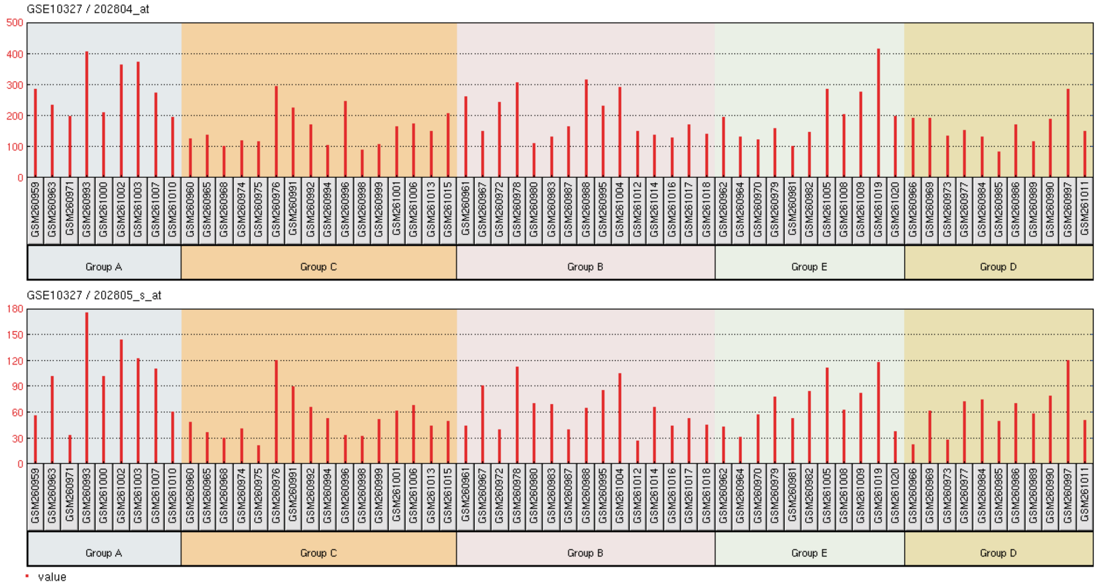

ABCC2

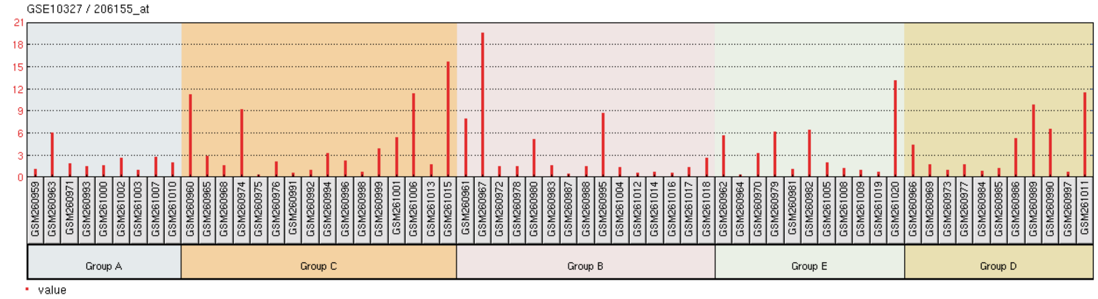

Supplement: Additional file 1 — Expression profiles for ABC transporters of particular interest, by molecular medulloblastoma subtype. Expression patterns from GEO2R analysis of dataset NCBI GEO GSE10327 (62 human medulloblastoma cases, organized by subtype), for ABCA1 and four key modulators of drug resistance in human cells (ABCG2, ABCB1, ABCC1 and ABCC2). [file 2162-3619-2-26-S1.pdf]
