# Supplementary material for: ABC transporter activity linked to radiation resistance and molecular subtype in pediatric medulloblastoma
Source: Exp Hematol Oncol. 2013 Oct 4;2:26. doi: 10.1186/2162-3619-2-26 (PMC3851566; doi:10.1186/2162-3619-2-26)

Ingram *et al.* Additional File 3:

Expression profiles for four ABC transporters that show highly significant medulloblastoma subtype associations.

Shown: ABC transporter family genes present in public dataset NCBI GEO GSE10327 (62 Meudulloblastoma cases), with combinatorial subtype discrimination p-values <0.000001 (see analysis in Supplementary File 2). X-axis scale = Affymetrix data signal strength value.

Group names are as in Kool *et al*., 2008. (Group A = WNT associated, Group B = SHH associated, while Group C + Group D = Group 4 and Group E = Group 3 in consensus nomenclature of Taylor et al. 2012)

Probeset 204719_at: ABCA8

Adj. p-value for discrimination of tumors of group B from ACDE = **0.000000003**

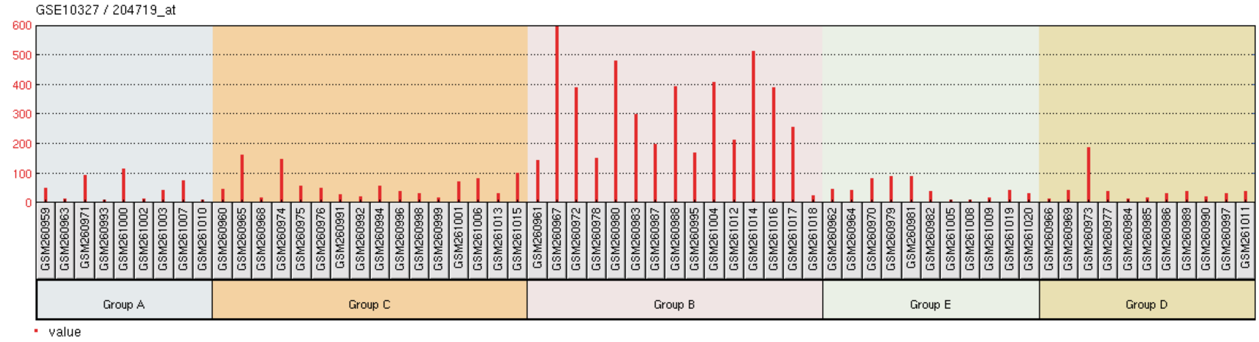

Probeset 210246_s_at: ABCC8

Adj. p-value for discrimination of tumours of group B from ACDE = **0.000000022**
Adj. p-value for discrimination of tumours of group BE from ACD = **0.000000006**

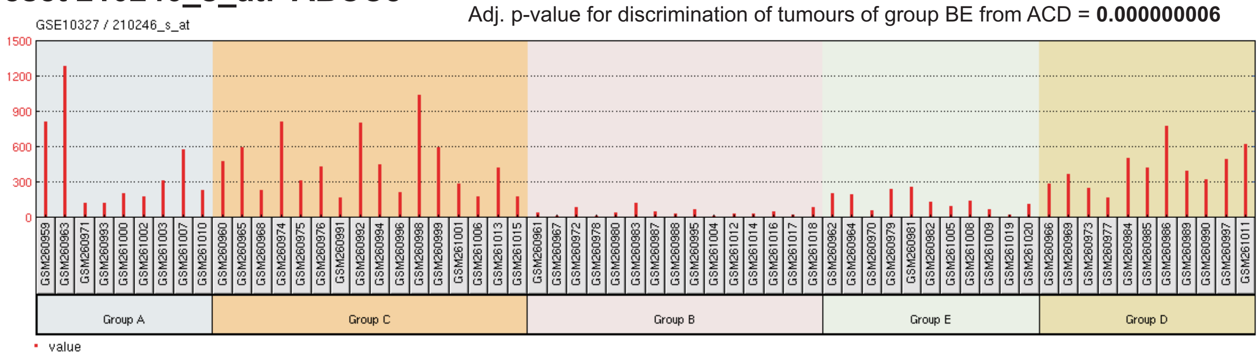

Probeset 207583_at: ABCD2

Adj. p-value for discrimination of tumours of group AB from CDE = **0.000000218**

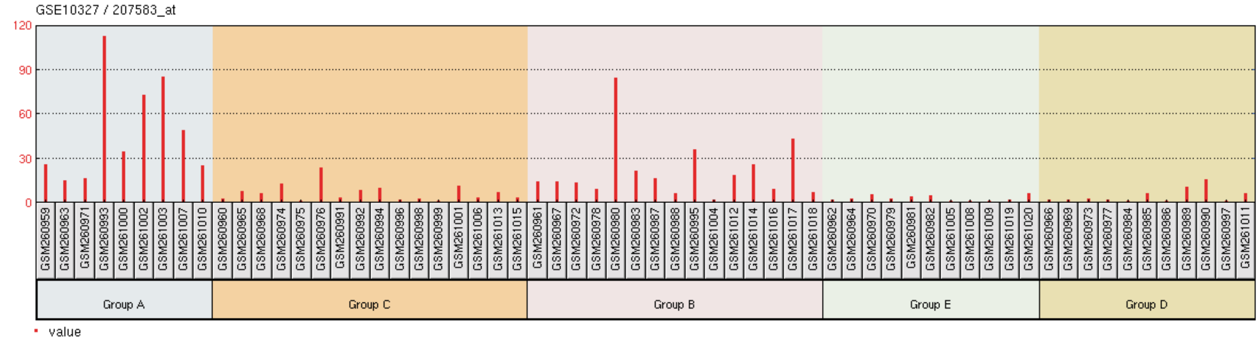

Probeset 207819_s_at: ABCB4

Adj. p-value for discrimination of tumors of group B from ACDE = **0.000000726**

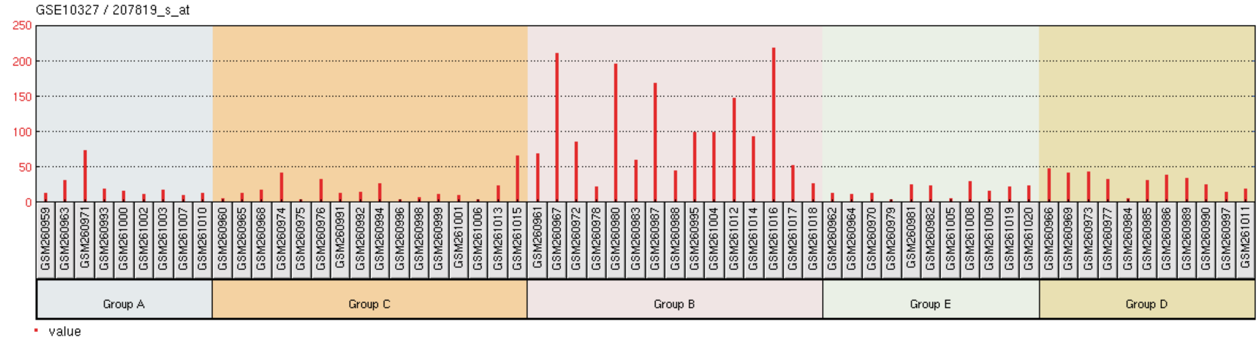

Supplement: Additional file 3 — Expression profiles for four ABC transporters that show highly significant medulloblastoma subtype associations. Expression patterns from GEO2R analysis of dataset NCBI GEO GSE10327 (62 human medulloblastoma cases), for ABC transporters found to have combinatorial subtype discrimination p-values < 0.000001 (ABCA8, ABCC8, ABCD2 and ABCB4). [file 2162-3619-2-26-S3.pdf]
