# Supplementary material for: ABC transporter activity linked to radiation resistance and molecular subtype in pediatric medulloblastoma
Source: Exp Hematol Oncol. 2013 Oct 4;2:26. doi: 10.1186/2162-3619-2-26 (PMC3851566; doi:10.1186/2162-3619-2-26)

Ingram *et al.*, Additional File 4: Medulloblastoma Gene Expression Heat Maps for ABC Transporter Family Members

Toronto Cohort

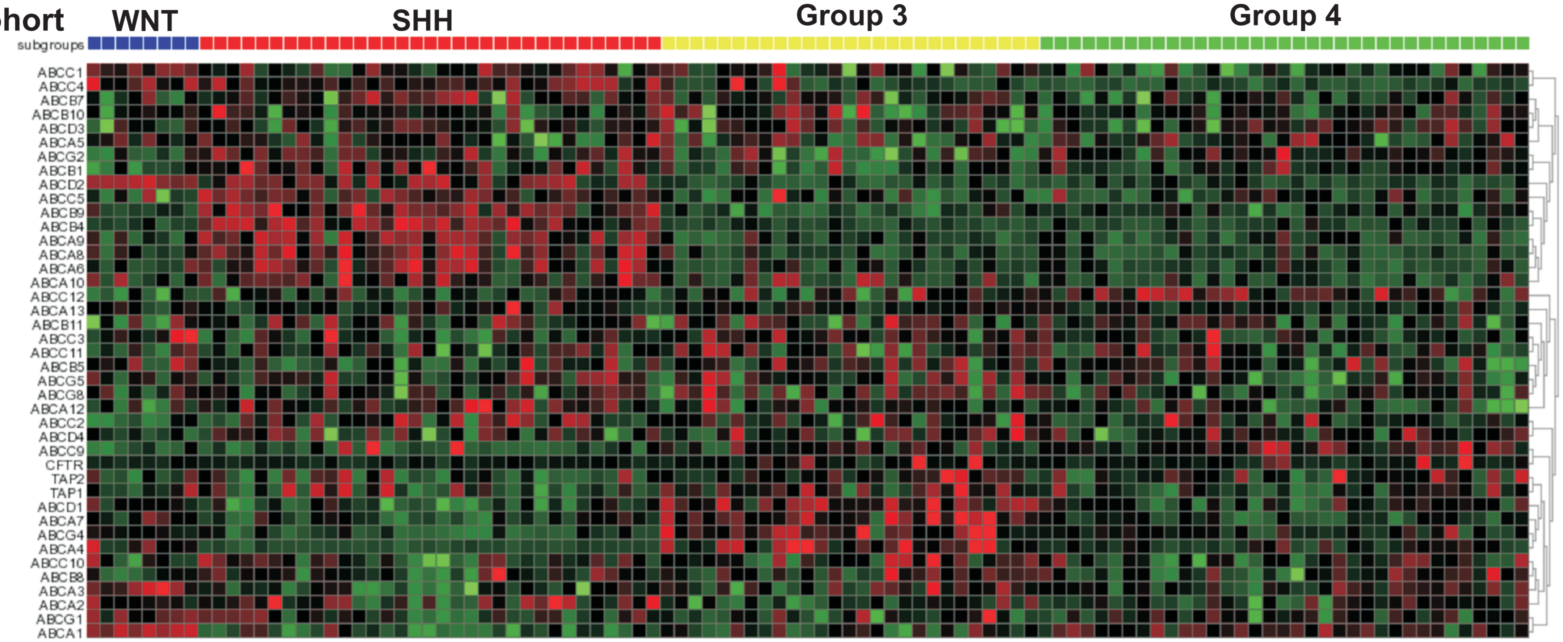

Boston Cohort

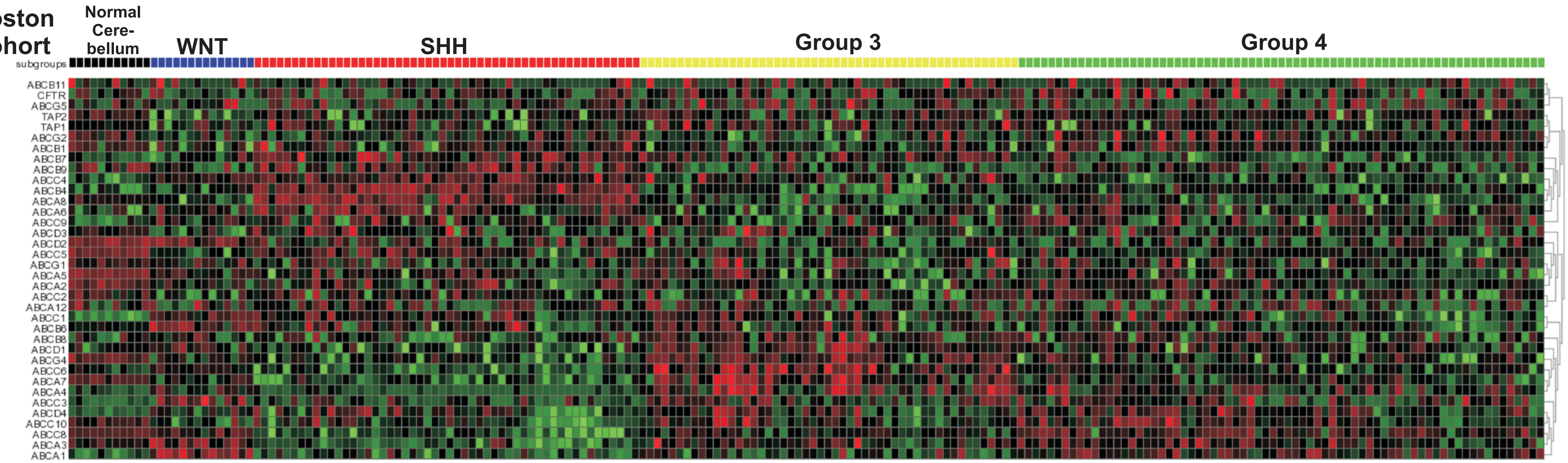

Supplement: Additional file 4 — Medulloblastoma Gene Expression Heat Maps for ABC Transporter Family Members. Expression data for available ABC Transporter genes in the Boston and Toronto sets, shown as heat maps (over-expression = red, under-expression = green). Patients are ordered by molecular subtype and clustered by ABC expression pattern. [file 2162-3619-2-26-S4.pdf]
