# Supplementary material for: ABC transporter activity linked to radiation resistance and molecular subtype in pediatric medulloblastoma
Source: Exp Hematol Oncol. 2013 Oct 4;2:26. doi: 10.1186/2162-3619-2-26 (PMC3851566; doi:10.1186/2162-3619-2-26)

Ingram *et al.*, Additional File 5:

ABCA1 is highly expressed in WNT driven medulloblastoma, relative to other subgroups and normal cerebellum

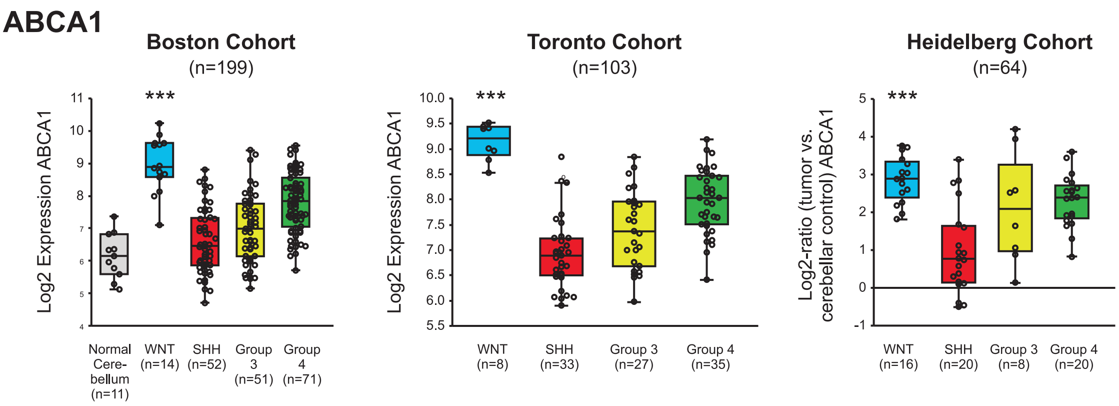

Supplement: Additional file 5 — ABCA1 is highly expressed in WNT driven medulloblastoma, relative to other subgroups and normal cerebellum.ABCA1 expression data from the three independent validation cohorts (n = 366 patients total), shown as box and whisker plots (quartiles and median indicated by box outline and centreline respectively). Asterisks indicate significance of difference in expression between the WNT tumors and the remaining subgroups (“***” = p-value <0.001). In addition, comparison between the WNT subgroup and normal cerebellum (available for the Boston cohort) showed a significant difference, with a p-value < 0.001. [file 2162-3619-2-26-S5.pdf]
